# Adolescent and Caregiver Perspectives on Family Navigation to Improve Healthcare Access and Use for Managing Pediatric Obesity

**DOI:** 10.1177/11786329231200863

**Published:** 2023-09-26

**Authors:** Geoff DC Ball, Marcus G O’Neill, Mitchell Rath, Maryam Kebbe, Arnaldo Perez, Ian Zenlea, Josephine Ho

**Affiliations:** 1 Department of Pediatrics, Faculty of Medicine & Dentistry, University of Alberta, Edmonton, AB, Canada; 2 Alberta Children’s Hospital, Department of Pediatrics, Cumming Faculty of Medicine, University of Calgary, Calgary, AB, Canada; 3 Reproductive Endocrinology and Women’s Health Laboratory, Pennington Biomedical Research Center, Louisiana State University, Baton Rouge, LA, USA; 4 School of Dentistry, Faculty of Medicine & Dentistry, University of Alberta, Edmonton, AB, Canada; 5 Institute for Better Health, Trillium Health Partners, Mississauga, ON, Canada

**Keywords:** Obesity, intervention, family navigation, interview, pediatric, family

## Abstract

We interviewed families to explore their views on the role of family navigation (FN) to improve access to and use of health services for managing pediatric obesity. From March to December, 2020, we conducted individual, structured telephone interviews with adolescents with obesity (13-17 years old) and their caregivers from Edmonton and Calgary, Canada. Among our 37 participants (14 adolescents, 23 caregivers), most (n = 27; 73.0%) reported FN could improve their access to obesity management. Participants recommended several activities to support healthcare access and use, including appointment reminders, evening/weekend appointments, parking/transportation support, and in-clinic childcare, all of which help families to attend appointments over an extended period to support obesity management. Most participants preferred FN be offered by healthcare professional ‘navigators’ who were approachable, empathic, and compassionate since issues regarding health and obesity can be sensitive, emotional topics to discuss. Overall, families supported integrating FN into multidisciplinary pediatric obesity management to improve healthcare access and use by navigators who apply a range of practical strategies and relational skills to enhance long-term access and adherence to care.

## Introduction

Pediatric obesity is common^1^ and often tracks into adulthood,^2^ which underscores the value of accessible, effective interventions to help children and adolescents improve their health and well-being.^3,4^ Effective behavioral interventions for managing pediatric obesity often require frequent clinic appointments^5-7^ and patient adherence to treatment plans over an extended period, including regular attendance at clinic appointments.^8^ However, achieving a high intervention dose and adequate access to care can be challenging for families due to barriers and constraints such as limited clinic schedule availability and high transportation costs.^9^

Individuals with a chronic illness, including obesity, often experience difficulties navigating the healthcare system because many health services are complex and fragmented.^10^ Patient or family navigation (FN) services were created in response to these difficulties to help individuals at risk of or with chronic illnesses to access therapeutic care. FN programs are usually offered by support workers (ie, patient or family navigators [“navigators”]) who supplement existing health services. Navigators help patients and their families to coordinate healthcare, access community services, attend clinical appointments, overcome communication and information challenges, and receive support and education.^11^ Given these roles, FN may help to improve healthcare access and use for families enrolled in multidisciplinary, pediatric obesity management, which may help to reduce attrition and optimize treatment outcomes.^12^ Our objective was to explore families’ views of the importance and role of FN to improve access to and use of health services for managing pediatric obesity. Data from this study informed a subsequent randomized controlled trial (RCT) to assess the feasibility and acceptability of FN to improve health services access and use as well as reduce attrition in pediatric obesity management.^13^

## Materials and Methods

Completed between March and December, 2020, we recruited a convenience sample of participants from hospital-based, multidisciplinary pediatric obesity management clinics in Edmonton and Calgary (Alberta, Canada). We chose to enroll families from these 2 clinics because our previous multi-center research^14^ showed that attrition was common and clinic leaders wanted to improve their health services to support family enrollment and participation. Adolescents (13-17 years old) were eligible to participate if they had an age- and sex-specific BMI ⩾ 85th percentile^15^ at the time of clinic referral; both active and discharged participants were eligible. Caregivers were eligible if they had a child (⩽17 years old) who was currently attending or had previously attended one of the clinics. Individuals were excluded if they did not speak English. Families were approached by their usual healthcare providers to describe the study and obtain consent to be contacted by the research team. Research team members (MGO, MR) verbally obtained informed assent and consent; all interactions with participants were virtual since our study was conducted during the COVID-19 pandemic. Each participant was offered a gift card ($10 CAD) as a token of appreciation. Our study was approved by research ethics boards at the University of Alberta and University of Calgary, with administrative approval provided by Alberta Health Services.

## Data Collection

Researchers interviewed participants by telephone (duration: 20-30 minutes) using a structured guide (see Supplemental Table). The guide was drafted and refined by our research team before implementation. Sociodemographic data were retrieved from adolescents’ medical records. Most study data were qualitative (interviews); quantitative data were collected to provide context and for descriptive purposes.

## Data Analysis

For our qualitative data, all interviews were audio-recorded and transcribed verbatim. To optimize accuracy and completeness, transcripts were cross-referenced using notes taken by researchers during the interviews. Manifest content analysis was used to identify perceived benefits, drawbacks, and preferred roles of FN for managing pediatric obesity. Sample quotes were chosen to support our qualitative analysis (ie, “A + number” represents a quote from an adolescent; “C + number” represents a quote from a caregiver). For our quantitative data, descriptive analyses (eg, means, frequencies) were calculated using *Microsoft Excel*. The same team members (MGO, MR) led our data analysis in consultation with our clinic/project leaders (GDCB, IZ, JH). Our study was not designed a priori to examine group differences between adolescents and caregivers, so no hypotheses were tested.

## Results

In total, 14 adolescents (8 males) and 23 parents (21 females) were enrolled and interviewed (total n = 37). Most adolescents and caregivers identified as white and had household incomes ⩾$100 000 (CDN) per year. All had attended clinical appointments for managing pediatric obesity; most were still attending appointments regularly (Table 1).

**Table 1.** Sociodemographic characteristics of study participants.

	Adolescents (n = 14)	Caregivers (n = 23)
Age (y)	15.5 ± 1.6	45.0 ± 7.0
Sex		
Male	n = 8, 57%	n = 2, 9%
Female	n = 6, 43%	n = 21, 91%
Height (cm)	173.1 ± 8.7	—
Weight (kg)	103.2 ± 24.2	—
BMI (kg/m^2^)	34.2 ± 6.3	—
BMI percentile	96.3 ± 5.6	—
BMI z-score	2.1 ± 0.6	—
Race/Ethnicity		
White	n = 8, 57%	n = 17, 74%
Non-white	n = 4, 29%	n = 4, 17%
Unknown	n = 1, 7%	n = 1, 4%
Prefer not to say	n = 1, 7%	n = 1, 4%
Residence		
Urban	n = 10, 71%	n = 18, 78%
Rural/remote	n = 4, 29%	n = 5, 22%
Clinic status		
Enrolled/active	n = 9, 64%	n = 13, 57%
Discharged	n = 5, 36%	n = 10, 43%
Annual household income (CAD)		
<$50 000	n = 1, 3%	
$50 000-100 000	n = 9, 24%	
>$100 000	n = 25, 68%	
Prefer not to say	n = 1, 3%	
Unknown	n = 1, 3%	

Data presented as mean ± standard deviation unless otherwise specified.

### Perceived benefits and drawbacks

Eleven (79%) adolescents and 16 (70%) caregivers agreed that navigators could improve their healthcare access and use of pediatric obesity management services. Most identified several benefits of FN. One caregiver indicated it would be helpful to have someone who could connect their family with community resources.

*We were new to the city. (It) would’ve been awesome (to have someone) to say, over in this area they offer this program or you know, you can access this.* (C8)

Another caregiver and an adolescent believed navigators could act as a go-between for them and the clinical team.

*If there is a conflict that arises (between the family and clinical team), someone that we can speak to outside of that, and they can help us resolve it.* (C10)

*I feel like they would’ve actually understood my feelings of being forced (to attend the clinic for obesity management).* (A1)

Although most participants had a favorable view of FN, some did not perceive benefits for them. Two caregivers reported:

*I don’t think it (a navigator) would have changed anything. I work in a healthcare field, so I know all the navigation. I know how to advocate. I know all of that.* (C7)

*This is such a small part of her overall medical needs that I think it would just add one more person that I’d have to talk to.* (C14)

Overall, 6 (43%) adolescents and 17 (74%) caregivers preferred navigators to work closely with the clinical team rather than having them function independently. One caregiver said:

*If (navigators) are close together that would be more benefit because they may have access to all kinds of information for the client that they (the multidisciplinary clinical team) have.* (C13)

One adolescent stated:

*I think that they shouldn’t be under the influence (of the clinical team) and they should try (to) go by the family’s way of doing things rather than the clinic’s.* (A12)

### Navigator characteristics

**Figure 1.**
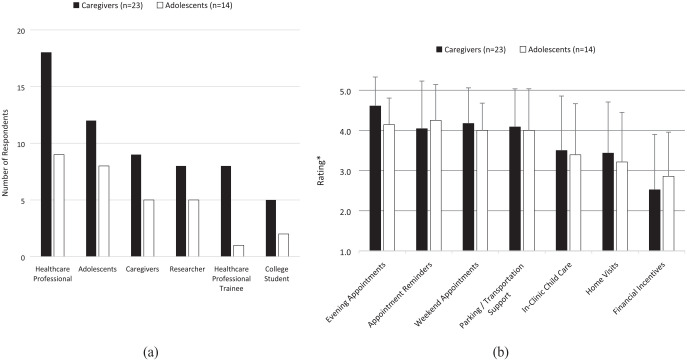
(a) Adolescent and caregiver preferences regarding navigator backgrounds and (b) adolescent and caregiver perceived importance of navigator activities. *Rating scale range: 1 = very unimportant; 2 = somewhat unimportant; 3 = neutral; 4 = somewhat important; 5 = very important.

Adolescents and caregivers reported similar preferences regarding navigator characteristics, including the following traits: “Approachable” (n = 14, 38%), “empathic” (n = 11, 30%), “compassionate” (n = 11, 30%), “good verbal communicator” (n = 10, 27%), and “knowledgeable” (n = 10, 27%). Participants also described their preferred navigator backgrounds (Figure 1a) and the activities navigators could offer to enhance access to pediatric obesity management (Figure 1b).

## Discussion

Most adolescents with obesity and caregivers believed FN would improve their healthcare access and use, although this view was not universal. The complexity of obesity underscores the varied resources and supports families need in managing pediatric obesity.^16^ Families with adequate resources (eg, social support, health literacy) may not perceive the need for FN, and those who believe that the barriers they face cannot be addressed (eg, lack of time, distance) by increasing their engagement in services. We did not query this issue directly, but FN programs appear to generate positive outcomes for vulnerable populations in particular.^17^ Identifying families with limited resources and tailoring the available support to families’ needs may be the most efficient and useful application of FN.

Adolescents and caregivers reported that FN programs could offer them several strategies to improve their healthcare access. Both practical (eg, text messaging) and relational (eg, motivational interviewing) strategies have been used to increase participation in pediatric obesity management, although the evidence of impact on treatment outcomes is limited.^12^ Participants preferred clinic appointments that were available at expanded times, including late afternoons, evenings, and weekends; these offerings can make it easier for families to access care while balancing work, school, and extra-curricular activities. Clinics that offer these appointment options may improve access and satisfaction with care^18^; however, limited data indicate that enhanced schedule availability independently enhances healthcare use or improves obesity-related outcomes. Preliminary findings suggest that families who self-refer into multidisciplinary, pediatric obesity management miss fewer clinic appointments than their clinician-referred peers.^19^ The current study included physician-referred families exclusively, but families may have better access to care, which could be enhanced by FN, if they could self-refer to services when they have adequate time, support, and interest.

Participants preferred navigators who possessed personal characteristics and applied strategies to enhance their access to care, which could be provided by a range of diverse individuals. Healthcare professionals were preferred (eg, nurses, dietitians). Qualitative analyses suggested that navigators’ characteristics may be the most important factors for effective, patient- and family-centered FN programs.^20^ Traits prioritized by adolescents and caregivers showed their preference for navigators with strong relational skills, which could be supplemented by advanced training in health coaching^21^ and motivational interviewing (MI).^22^ Clinical practice guidelines emphasize the potential value for MI to support families in managing pediatric obesity.^23^

Our study was not without limitations. First, participant recruitment for our study was impacted negatively by the COVID-19 pandemic. We enrolled as many participants as possible during our 10-month study period, but our pool of potential participants was low due to pandemic-related restrictions and the redeployment of clinical team members to complete high priority tasks in our hospitals (eg, patient screening). This issue reduced the total number of participants we could recruit; it may have also limited the sociodemographic diversity (eg, culture/ethnicity, socioeconomic status) of our sample since restrictions during the COVID-19 pandemic impacted health care access and use,^24^ which were lower in individuals with higher levels of socioeconomic deprivation or among populations of visible minorities. These data demonstrated how COVID-19 exacerbated existing health inequities and likely reduced study participation for families that could benefit the most from FN. Second, neither of our 2 clinics included FN, so we were only able to assess adolescents’ and caregivers’ perceived preferences. To our knowledge, FN has not been applied or evaluated as a strategy to improve health care access and use or reduce attrition in pediatric obesity management. The novelty of FN meant that we could not explore families’ experiences with FN, which may differ from their preferences.

Overall, most families had a favorable view of FN and believed navigators could improve healthcare access and use in pediatric obesity management. Data from this study informed an RCT by our team to assess FN feasibility and acceptability,^13^ so we will generate original data (eg, families’ experiences, acceptability, health care use, attrition) that can support decisions related to how and by whom health services are offered for adolescents and their families managing pediatric obesity.

## Supplemental Material

sj-docx-1-his-10.1177_11786329231200863 – Supplemental material for Adolescent and Caregiver Perspectives on Family Navigation to Improve Healthcare Access and Use for Managing Pediatric Obesity Click here for additional data file. Supplemental material, sj-docx-1-his-10.1177_11786329231200863 for Adolescent and Caregiver Perspectives on Family Navigation to Improve Healthcare Access and Use for Managing Pediatric Obesity by Geoff DC Ball, Marcus G O’Neill, Mitchell Rath, Maryam Kebbe, Arnaldo Perez, Ian Zenlea and Josephine Ho in Health Services Insights

sj-docx-2-his-10.1177_11786329231200863 – Supplemental material for Adolescent and Caregiver Perspectives on Family Navigation to Improve Healthcare Access and Use for Managing Pediatric Obesity Click here for additional data file. Supplemental material, sj-docx-2-his-10.1177_11786329231200863 for Adolescent and Caregiver Perspectives on Family Navigation to Improve Healthcare Access and Use for Managing Pediatric Obesity by Geoff DC Ball, Marcus G O’Neill, Mitchell Rath, Maryam Kebbe, Arnaldo Perez, Ian Zenlea and Josephine Ho in Health Services Insights

sj-docx-3-his-10.1177_11786329231200863 – Supplemental material for Adolescent and Caregiver Perspectives on Family Navigation to Improve Healthcare Access and Use for Managing Pediatric Obesity Click here for additional data file. Supplemental material, sj-docx-3-his-10.1177_11786329231200863 for Adolescent and Caregiver Perspectives on Family Navigation to Improve Healthcare Access and Use for Managing Pediatric Obesity by Geoff DC Ball, Marcus G O’Neill, Mitchell Rath, Maryam Kebbe, Arnaldo Perez, Ian Zenlea and Josephine Ho in Health Services Insights

## References

[1] Rodd C Sharma AK . Recent trends in the prevalence of overweight and obesity among Canadian children. Can Med Assoc J. 2016;188:E313-E320. 10.1503/cmaj.150854 PMC5026530 27160875

[2] Cunningham SA Datar A Narayan KMV Kramer MR . Entrenched obesity in childhood: findings from a national cohort study. Ann Epidemiol. 2017;27:435-441. 28645567 10.1016/j.annepidem.2017.05.016 PMC5550333

[3] Al-Khudairy L Loveman E Colquitt JL , et al. Diet, physical activity and behavioural interventions for the treatment of overweight or obese adolescents aged 12 to 17 years. Cochrane Database Syst Rev. 2017;6:CD012691. 10.1002/14651858.CD012691 PMC6481371 28639320

[4] Mead E Brown T Rees K , et al. Diet, physical activity and behavioural interventions for the treatment of overweight or obese children from the age of 6 to 11 years. Cochrane Database Syst Rev. 2017;6:CD012651. 10.1002/14651858.CD012651 PMC6481885 28639319

[5] Wilfley DE Staiano AE Altman M , et al. Improving access and systems of care for evidence-based childhood obesity treatment: conference key findings and next steps. Obesity (Silver Spring). 2017;25:16-29. 27925451 10.1002/oby.21712 PMC5373656

[6] Hoedjes M Makkes S Halberstadt J , et al. Health-related quality of life in children and adolescents with severe obesity after intensive lifestyle treatment and at 1-year follow-up. Obes Facts. 2018;11:116-128. 29631271 10.1159/000487328 PMC5981677

[7] Reinehr T Lass N Toschke C Rothermel J Lanzinger S Holl RW . Which amount of BMI-SDS reduction is necessary to improve cardiovascular risk factors in overweight children? J Clin Endocrinol Metab. 2016;101:3171-3179. 27285295 10.1210/jc.2016-1885

[8] Boff RM Liboni RPA Batista IPA de Souza LH Oliveira MDS . Weight loss interventions for overweight and obese adolescents: a systematic review. Eat Weight Disord. 2017;22:211-229. 27542161 10.1007/s40519-016-0309-1

[9] Dhaliwal J Nosworthy NMI Holt NL , et al. Attrition and the management of pediatric obesity: an integrative review. Child Obes. 2014;10:461-473. 25496035 10.1089/chi.2014.0060

[10] Levesque JF Harris MF Russell G . Patient-centred access to health care: conceptualising access at the interface of health systems and populations. Int J Equity Health. 2013;12:18. 23496984 10.1186/1475-9276-12-18 PMC3610159

[11] McBrien KA Ivers N Barnieh L , et al. Patient navigators for people with chronic disease: a systematic review. PLoS One. 2018;13:e0191980. 10.1371/journal.pone.0191980 PMC5819768 29462179

[12] Ball GDC Sebastianski M Wijesundera J , et al. Strategies to reduce attrition in managing paediatric obesity: a systematic review. Pediatr Obes. 2021;16:e12733. 10.1111/ijpo.12733 32959990

[13] Ball GDC O’Neill MG Noor R , et al. A multi-center, randomized, 12-month, parallel-group, feasibility study to assess the acceptability and preliminary impact of family navigation plus usual care versus usual care on attrition in managing pediatric obesity: a study protocol. Pilot Feasibility Stud. 2023;9:14. 36691103 10.1186/s40814-023-01246-w PMC9868519

[14] Morrison KM Ball GDC Ho J , et al. The CANadian Pediatric Weight management Registry (CANPWR): lessons learned from developing and initiating a national, multi-centre study embedded in pediatric clinical practice. BMC Pediatr. 2018;18:237. 30025530 10.1186/s12887-018-1208-6 PMC6053829

[15] Dietitians of Canada, Canadian Paediatric Society, The College of Family Physicians of Canada, Community Health Nurses of Canada, Secker D . Promoting optimal monitoring of child growth in Canada: using the new WHO growth charts. Can J Diet Pract Res. 2010;71:e1-e3. 10.3148/71.1.2010.54 21815309

[16] Avis JL Bridger T Buchholz A , et al. It’s like rocket science. . .only more complex: challenges and experiences related to managing pediatric obesity in Canada. Expert Rev Endocrinol Metab. 2014;9:223-229. 30736161 10.1586/17446651.2014.897605

[17] Budde H Williams GA Winkelmann J Pfirter L Maier CB . The role of patient navigators in ambulatory care: overview of systematic reviews. BMC Health Serv Res. 2021;21:1166. 34706733 10.1186/s12913-021-07140-6 PMC8555047

[18] McMaster CM Cohen J Alexander S , et al. Satisfaction and acceptability of paediatric weight management services amongst parents and carers: a mixed-methods study. Clin Obes. 2020;10:e12391. 10.1111/cob.12391 32830905

[19] McGeown L Ball GDC Mushquash AR . Is there a role for self-referral in pediatric weight management? Child Obes. 2021;17:559-562. 34415796 10.1089/chi.2021.0161

[20] Peart A Lewis V Barton C Russell G . Healthcare professionals providing care coordination to people living with multimorbidity: an interpretative phenomenological analysis. J Clin Nurs. 2020;29:2317-2328. 32221995 10.1111/jocn.15243

[21] Schwellnus H King G Thompson L . Client-centred coaching in the paediatric health professions: a critical scoping review. Disabil Rehabil. 2015;37:1305-1315. 25289943 10.3109/09638288.2014.962105

[22] Kaczmarek T Kavanagh DJ Lazzarini PA Warnock J Van Netten JJ . Training diabetes healthcare practitioners in motivational interviewing: a systematic review. Health Psychol Rev. 2022;16:430-449. 33970799 10.1080/17437199.2021.1926308

[23] Hampl SE Hassink SG Skinner AC , et al. Executive summary: clinical practice guideline for the evaluation and treatment of children and adolescents with obesity. Pediatrics. 2023;151:e2022060640. 10.1542/peds.2022-060641 36622135

[24] Warner M Burn S Stoye G Aylin PP Bottle A Propper C . Socioeconomic deprivation and ethnicity inequalities in disruption to NHS hospital admissions during the COVID-19 pandemic: a national observational study. BMJ Qual Saf. 2022;31:590-598. 10.1136/bmjqs-2021-013942 PMC8627367 34824162

