# Supplementary material for: Adolescent and Caregiver Perspectives on Family Navigation to Improve Healthcare Access and Use for Managing Pediatric Obesity
Source: Health Serv Insights. 2023 Sep 26;16:11786329231200863. doi: 10.1177/11786329231200863 (PMC10524045; doi:10.1177/11786329231200863)
Supplement: sj-docx-1-his-10.1177_11786329231200863 – Supplemental material for Adolescent and Caregiver Perspectives on Family Navigation to Improve Healthcare Access and Use for Managing Pediatric Obesity [file sj-docx-1-his-10.1177_11786329231200863.docx]

**Highlights**

- Many families face challenges in accessing care for managing pediatric obesity, limiting treatment impact.
- Family navigation (FN) enhances healthcare access and use for patients and families managing chronic illnesses.
- Most adolescents and caregivers in our study reported that FN could improve their access to and use of health services for managing obesity, with navigators applying diverse strategies and skills.
