# Supplementary material for: Adolescent and Caregiver Perspectives on Family Navigation to Improve Healthcare Access and Use for Managing Pediatric Obesity
Source: Health Serv Insights. 2023 Sep 26;16:11786329231200863. doi: 10.1177/11786329231200863 (PMC10524045; doi:10.1177/11786329231200863)
Supplement: sj-docx-3-his-10.1177_11786329231200863 – Supplemental material for Adolescent and Caregiver Perspectives on Family Navigation to Improve Healthcare Access and Use for Managing Pediatric Obesity [file sj-docx-3-his-10.1177_11786329231200863.docx]

**Supplemental Table. Interview guide**

| 1. We want to know whether you believe it is helpful for families to have someone at the clinic who helps them access support and help. We are calling this person a ‘family navigator’. The navigator will have a specific role to help support families like you. Do you think your family would benefit (or have benefited) from a navigator?    1. If “yes”, how do you think a navigator might be helpful? What kinds of things would you want a family navigator to help you and your family with?    2. If “no”, what would help you and your family to access support from the clinic? How might a family navigator be helpful for other families? |
| --- |
| 1. In your opinion, do you think it would be better for the family navigator to be part of (and work closely with) the health care team at the clinic **or** do you think it would be better for the family navigator to a bit separate from the health care team? Please explain. |
| 1. Several people could act as family navigators. Of the following, which ones do you think would offer you and your family with the best support as a family navigator (select all that apply):    1. Health care professional (*e.g.,* nurse, social worker, dietitian)    2. Adolescent who previously participated in the clinic    3. Parent of child who previously participated in the clinic    4. Health care professional ‘in-training’    5. College or university student    6. Research assistant |
| 1. What personal characteristics should a family navigator possess that will make him/her most helpful to you and your family? |
| 1. Several things can make it easier for families to access support and help from the clinic. Often, family navigators have a variety of resources available to them. Of the following things, please rate how important they are to helping you and your family access care from the clinic (1 = very unimportant; 2 = somewhat unimportant; 3 = neutral; 4 = somewhat important; 5 = very important):    1. Late afternoon or early evening clinic appointments    2. Appointment reminders (text, phone and/or email)    3. Parking and transportation support    4. Weekend clinic appointments    5. Financial incentive    6. In-clinic childcare    7. Home visits |
